# Selenium + Vitamin E Supplementation in Anestrus Goats: A Strategy to Enhance Reproductive Outcomes Under a Semi-Arid Production System

**DOI:** 10.3390/ani15101421

**Published:** 2025-05-14

**Authors:** Raquel Santos-Silva, Cesar A. Meza-Herrera, Brenda Castro-Roque, Guadalupe Calderón-Leyva, Cayetano Navarrete-Molina, Oscar Angel-García, Jessica M. Flores-Salas, Angeles De Santiago-Miramontes, Fernando Arellano-Rodriguez, Cesar A. Rosales-Nieto

**Affiliations:** 1 Graduate Program—Natural Resources and Environment in Arid Lands, Regional Universitary Unit on Arid Lands, Chapingo Autonomous University, Bermejillo, Durango 35230, Mexico; 2 Graduate Program—Agricultural and Livestock Sciences, Antonio Narro Agricultural Autonomous University, Laguna Unit, Torreon Coahuila 27054, Mexico; 3 Department of Chemistry, Environmental Technology Area, Rodeo Technological University, Rodeo, Durango 35760, Mexico; 4 Department of Agricultural Sciences, Texas State University, San Marcos, TX 78666, USA

**Keywords:** goats, anestrous, male effect, targeted supplementation, reproductive efficiency, food security, zero hunger

## Abstract

Goats are primarily raised in marginal, arid, and semi-arid production systems and exhibit a seasonal reproductive pattern. Extending their reproductive season would enhance food security and improve the economic viability of goat production. Therefore, the objective of this study was to assess the effects of SeVE supplementation during the anestrus season on ovarian activity in anestrous goats subjected to an estrus synchronization protocol. The response variables included estrus induction (EI), ovulatory rate (OR), corpus luteum diameter (CLD), left and right ovary diameters (LOD and ROD), embryo implantation (EMBRYO), and pregnancy rates (PREG). Adult, anestrous, crossbred dairy goats (*n* = 32) from northern semi-arid Mexico (i.e., Comarca Lagunera, 26° N) were randomly assigned to two homogeneous groups based on age, live weight (LW), and body condition score (BCS): (1) the supplemented group (SeVE; *n* = 16), received 5 mg selenium plus 350 IU vitamin E, and (2) the control group (CONT; *n* = 16), received physiological saline. Estrus was induced using a combination of progesterone (P4, 20 mg), equine chorionic gonadotropin (eCG, 400 IU), and exposure to the male effect. While no differences (*p* > 0.05) were observed between the groups for LW and BCS, the variables EI, OR, EMBRYO, and PREG rates were higher (*p* < 0.05) in the SeVE group than the CONT group. No differences (*p* > 0.05) were observed between the groups for CLD, LOD, or ROD. Thus, SeVE-targeted supplementation improved reproductive outcomes without affecting ovarian structure size (i.e., a non-dimensional ovarian SeVE action), suggesting that this precision supplementation strategy certainly enhances reproductive efficiency. This was possibly related to selenium-induced increases in P4 synthesis, and the antioxidant effects on follicular development correlate with improved endometrial development, conceptus survival, embryo growth, and pregnancy maintenance. Such improvements could contribute to the sustainability of marginal production systems in the semi-arid regions of northern Mexico, supporting food security and increasing economic returns for goat producers and their families.

## Simple Summary

Seasonal reproduction in goats leads to production seasonality, presenting physiological and economic challenges for producers who aim to extend the reproductive season to ensure a more consistent supply of products and year-round economic returns. Within this complex biological, physiological, and economic context, precision nutritional supplementation to enhance out-of-season ovarian activity in females appears to be a promising strategy. This study evaluated the possible effect of selenium (5 mg) plus vitamin E (350 IU) supplementation (i.e., SeVE) on ovarian activity, embryo implantation, and pregnancy rates in estrus-induced anestrous goats. SeVE supplementation increased (*p* < 0.05) estrus induction, ovulatory rate, embryo implantation, and pregnancy rates compared to the control group, without affecting (*p* > 0.05) corpus luteum diameter or left and right ovary diameters.

## 1. Introduction

Goat production is a vital agricultural activity that has contributed significantly to human development, particularly in marginal, arid, and semi-arid ecosystems and rural communities facing poverty. In these regions, goats provide employment opportunities and a critical source of animal protein for families, often representing their primary or sole source of income [1,2,3,4]. Goats exhibit remarkable adaptability across diverse agroecological zones, owing to their ability to withstand droughts and extreme climates, combined with desirable traits such as high prolificacy, rapid growth rates, and efficient utilization of low-quality plant resources [2,3]. In northern arid Mexico, goat production remains a cornerstone of rural economies, accounting for a substantial proportion of farmers’ income [5]. As of 2023, Mexico reported a goat population of approximately 9 million head, with the Comarca Lagunera (i.e., CL; 26° N) standing out as the leading goat-producing agroecological region, housing 404,414 goats [6]. The CL, a semi-arid region, is recognized nationally for its high goat milk production. However, pronounced seasonal fluctuations in vegetation and crop residue availability have resulted in a seasonal reproductive pattern, subsequently driving a seasonal production cycle for both milk and meat [7].

This reproductive seasonality imposes biological constraints that limit the sustainability of marginal goat production systems [8]. Goat reproduction is primarily regulated by photoperiodic variations, with a period of reproductive quiescence—marked by the absence of estrus and ovulatory activity—extending from January to early June. Reproductive activity typically resumes in late June and continues through December [9]. This pattern presents a significant physiological challenge for goat farmers seeking to extend the reproductive season to ensure more continuous production [10]. In this complex biological framework, the strategic use of socio-sexual cues (i.e., the male effect) combined with precision nutritional supplementation can enhance the metabolic status of females and facilitate the reactivation of the hypothalamic–pituitary–gonadal axis, thereby inducing out-of-season ovarian activity [10]. Consequently, there is an urgent need to identify targeted supplementation strategies to boost reproductive efficiency [11].

The use of vitamins and minerals has emerged as a promising supplementation strategy to enhance animal productivity and reproductive performance, given their role as enzymatic cofactors in multiple metabolic pathways [12]. Selenium (Se) and vitamin E (VE), in particular, act synergistically to exert antioxidant effects through independent biochemical mechanisms [13]. These antioxidants protect cellular membranes from oxidative damage induced by reactive oxygen species and lipid hydroperoxides [14], thereby preserving the integrity of DNA, RNA, and proteins, and preventing cell death by reducing oxidative stress [15]. Improvements in growth, reproductive performance, immune competence, hormone synthesis, and tissue integrity have been associated with adequate Se and VE status [14]. Specifically, sufficient blood Se levels have been linked to enhanced progesterone secretion [16], improved corpus luteum development [17], and reduced lipoperoxide concentrations [18]. Moreover, Se and selenoproteins (i.e., SeProt) are involved in lipid metabolism (i.e., cholesterol-to-progesterone) due to their ability to modulate redox homeostasis. In the last processes, some SeProt encoding genes (i.e., GPX1, GPX4, SELENOP, and SELENOF) and the redox-related gene (i.e., SOD2) were involved [19]. Additionally, SeVE supplementation has been shown to improve ovarian function, mitigate morphological changes, and minimize oxidative damage to ovaries and follicles [20,21,22,23]. SeVE has also been associated with a shortened postpartum estrus interval [17] and plays a crucial role in the activation of key enzyme systems such as glutathione peroxidase (GSH-Px) and deiodinases, both essential for optimal embryonic development [24]. These effects collectively contribute to higher pregnancy rates and increased kidding percentages [25]. Given that oxidative stress during seasonal anestrus may further suppress ovarian activity, the antioxidant properties of SeVE could mitigate this constraint by enhancing both follicular resilience and pregnancy rates. Besides, and from a social and environmental standpoint, enhancing goat reproductive efficiency aligns with several Sustainable Development Goals (SDGs), notably contributing to food security (SDG 2) and promoting responsible production practices (SDG 12) [2,26,27]. Building on these previous findings, we hypothesized that precision supplementation with SeVE would improve out-of-season reproductive performance in adult anestrus crossbred dairy goats in northern semi-arid Mexico, leading to increased ovarian activity, embryo implantation, and pregnancy rates. The present study was designed to test this hypothesis.

## 2. Materials and Methods

### 2.1. General

All experimental procedures, animal handling, and management practices in this study were conducted strictly within international [28] and national [29] guidelines for the ethical treatment, care, and welfare of research animals. While all the reported procedures were aligned with conventional veterinary practices, the study received institutional approval under the reference number UAAAN-UL-38111-425501002-2876.

### 2.2. Location and Environmental Conditions

The study was conducted in the ejido Lázaro Cárdenas, San Pedro, Coahuila de Zaragoza, Mexico (25°45′50″ N, 103°11′03″ W; altitude 1111 m above sea level). The region is characterized by a very dry, semi-warm climate, with rainfall concentrated between May and October; the annual precipitation ranges from 200 to 300 mm [30,31]. Average annual temperatures are approximately 24 °C, with summer highs reaching 41 °C and winter lows dropping to −1 °C. The study was carried out in two phases: (1) the male-to-female interaction phase during April and May, corresponding to the natural seasonal anestrus period, and (2) the implantation, embryogenesis, and early gestation phase during June and July.

### 2.3. Animal Management and Rangeland Conditions

**Figure 1:**
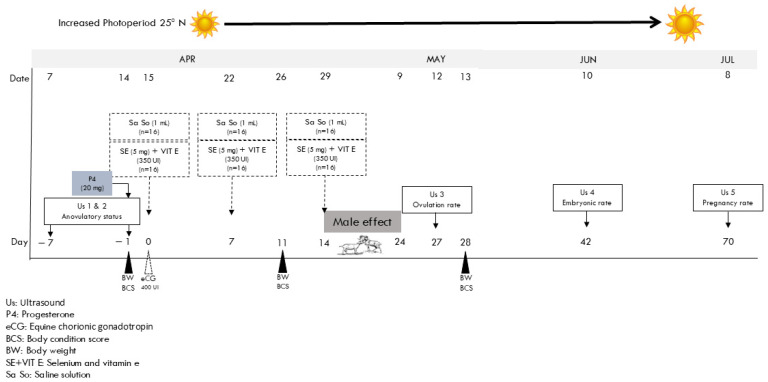
A schematic overview of the experimental timeline is presented. Two ultrasounds (US 1 and 2) were performed before estrus induction (following P4 and eCG administration), and subsequent evaluations (US 3, 4, and 5) assessed ovulation rate, ovarian activity, embryo development, and pregnancy status during the seasonal anestrus period at 25° N latitude.

Multiparous, non-lactating, non-pregnant, anestrous, crossbred goats (i.e., Criollo × Alpine–Saanen–Nubian; *n* = 32) of known fertility were selected from a 160-goat herd managed under a interesting semi-intensive production system subdivided according to their stage of lactation. In this peculiar production system, those goats that had just given birth and those in the 1/3 and 2/3 lactation (i.e., ≈9 months on lactation) were kept under an intensive management scheme (i.e., zero grazing) with free access to alfalfa hay, mineral salts, and water, while receiving 200 g day^−1^ goat^−1^ of a commercial concentrate (14% CP) (see Table 1). On the other hand, the goats in the 3/3 period of lactation, or all through their dry period, were kept under a semi-intensive management approach consisting of a diurnal grazing scheme with evening confinement with reduced nutritional support. In both corrals/groups, the goats were hand-milked daily in the morning; in the second group, milking was carried out before grazing. Grazing was allowed from 1000 to 1800 h in the grazing areas surrounding the production unit, with the guidance of a herdsman. The rangeland has flora characterized as Chihuahuan desert rangeland; while creosote bush (*Larrea tridentata* (DC. Cov)) dominates the grazing area, different key species consist of lechuguilla (*Agave lechuguilla* Torr), mesquite (*Prosopis glandulosa* v. glandulosa), and blue gramma (*Bouteloua gracilis* (Wild). Ex Kunth Lag. Ex Griffiths). Goats walk approximately 5–8 km daily from the pen to diverse sites of the available rangeland, thus grazing restraints can be considered negligible. Once in the pen, after the grazing period, all 32 goats received 200 g of alfalfa hay and 100 g of a commercial concentrate (14% CP), day^−1^ goat^−1^, with free access to mineral salts and water (see Table 1), during the whole experimental period. Since all the goats received such nutritional support on an individual basis and grazed the same rangeland, the goats were considered the experimental unit. The selected goats were homogeneous regarding live weight (LW; 39.4 ± 1.3 kg), body condition score (BCS 1.7 ± 0.0 units, scale: 1 = very thin to 4 = very fat), and age (2.4 ± 0.3 years old) to ensure balanced groups before randomization. To confirm anovulatory status during the increasing photoperiod (April, Northern Hemisphere; Figure 1), ovarian activity was evaluated by an expert using two transrectal ultrasound examinations (Aloka SSD 500, Tokyo, Japan, 7.5 MHz transducer) conducted seven days apart [32,33].

### 2.4. Experimental Procedures, Experimental Groups, and Treatment Design

Goats showing no evidence of follicular development (*n* = 32) were selected for estrus induction. Estrus was induced via intramuscular injection of 20 mg progesterone (P4; Progestelas “E”, Lab Aranda, Mexico City, Mexico), followed 24 h later by 400 IU equine chorionic gonadotropin (eCG; Foligon, MSD, Animal Health, Mexico City, Mexico) administered intramuscularly. This was designated as Day 0 of the experimental protocol. Then, goats were randomly assigned to two experimental groups: Supplemented group (SeVE; *n* = 16) received three intramuscular applications of 5 mg selenium plus 350 IU vitamin E (i.e., SELEBEN-B, Maymo Lab., S.A., Madrid, Spain) administered at 7-day intervals. Control group (CONT; *n* = 16), received three intramuscular applications of 1 mL physiological saline solution at 7-day intervals. Following treatments, sexually active males were introduced to both experimental groups for 10 days to induce estrus via the “male effect” (Figure 1) [35,36].

### 2.5. Measurements and Response Variables

#### 2.5.1. Corporal Measurements

Live weight (LW) and body condition score (BCS) were recorded at three points: at the beginning of the experiment, 12 days after initiation, and one day after the third ultrasound evaluation. Body condition was assessed by palpating the lumbar region and scoring on a scale from 1 (very thin) to 4 (very fat).

#### 2.5.2. Determination of Ovarian Size, Structures, and Activity

Thirteen days after male introduction, a transrectal ultrasound (Aloka SSD 500, Tokyo, Japan, 7.5 MHz transducer) was performed to assess ovarian structures in each goat. Estrus induction was determined by calculating the percentage of goats presenting a corpus luteum (i.e., CL) relative to the total number of goats per group. Additionally, the number and diameter of corpora lutea and the left and right ovary diameters were recorded.

#### 2.5.3. Embryonic Rate and Pregnancy Percentage

At 28 days post-male introduction, a fourth ultrasound (Aloka SSD 500, Tokyo, Japan, 7.5 MHz transducer) examination was conducted to detect embryonic vesicles and quantify the number of embryos per female. Embryo implantation rate was calculated as the number of embryos divided by the number of ovulated females per group. Pregnancy confirmation was performed at 56 days post-male introduction via a fifth ultrasound evaluation.

### 2.6. Statistical Analyses

At enrollment, goats were blocked based on LW and BCS. The response variables of attributes such as LW and BCS were analyzed by a completely random design split-plot analysis of variance for repeated samples in the same animal across time. Response variables included estrus induction response (EI), corpus luteum number (OR) and diameter (CLD), left and right ovary diameter (LOD and ROD), embryonic implantation rate (EMBRYO), and pregnancy rate (PREG). Since both treatment application and response variable quantification were individually collected in both experimental groups, the goat was considered the experimental unit. Thus, the fixed effects treatments as well as sampling date were assessed using a MIXED model for repeated measures across time, with time as the repeated measure and treatment as the repeated subject, regarded as the random error term. Estrus induction (i.e., induced or not) as well as pregnancy rate (i.e., pregnant or not) were analyzed using the generalized linear mixed model procedures with a binomial distribution and logit link function (PROC-GLIMMIX); treatment was the fixed effect. Least-squares means and standard errors for each class of treatments, sampling time, and their interaction were computed; mean comparisons were solved by means of Fisher’s least significant difference. All statistical analyses were done using the procedures of the SAS statistical package version 9.2; a significant difference between means was set at *p* < 0.05.

## 3. Results

Table 2 summarizes both experimental groups’ corporal, ovarian, and reproductive responses. No differences (*p* > 0.05) were found between groups for initial live weight (LW; 41.78 ± 1.08 kg) and body condition score (BCS; 1.69 ± 0.04 units) at the beginning of the breeding period. Similarly, no differences (*p* > 0.05) were observed for corpus luteum diameter (CLD; 9.1 ± 1.5 mm), left ovary diameter (LOD; 16.6 ± 1.4 mm), or right ovary diameter (ROD; 16.3 ± 1.3 mm). However, key reproductive variables—estrus induction (%), ovulation rate (units), number of embryos (units), and pregnancy rate (%)—were higher (*p* < 0.05) in the SeVE-supplemented group compared to the control group. Specifically, the SeVE group exhibited increases of 43.75%, 35.00%, 28.50%, and 41.33% over the control group values, respectively. These results indicate that SeVE supplementation improved ovarian activity (i.e., ovulation rate) and enhanced reproductive outcomes (i.e., estrus induction, embryo implantation, and pregnancy rate), without affecting ovarian structure size, suggesting a non-dimensional morphological SeVE’s action.

## 4. Discussion

Globally, goat production represents a critical source of income and food security for smallholders, particularly in arid and semi-arid regions where other livestock species face significant production constraints. This study tested the hypothesis that SeVE supplementation enhances reproductive performance by increasing ovarian activity, embryo implantation rates, and pregnancy rates in crossbred goats during the anestrus season. The results support this hypothesis: SeVE-supplemented goats exhibited higher values for key reproductive variables without differences in live weight, body condition, or ovarian diameter relative to controls. Therefore, our working hypothesis is not rejected. Interestingly, despite these reproductive enhancements, ovarian structures and corpus luteum diameters remained unaffected. These findings highlight SeVE supplementation as a viable strategy for advancing sustainable livestock production and contributing to global development goals related to responsible production and food security.

At the onset of the experiment, anestrus status was confirmed in all the goats. Following treatment, the SeVE group achieved an 80% estrus induction rate, consistent with previous studies using 10–20 mg P4 and 100 IU eCG administered 24 h after P4 in goats during the anestrus season [37]. The administration of eCG after P4 appears to shorten the time to follicular growth, likely mimicking the luteinizing hormone (LH) surge [38,39,40]. Moreover, a 20 mg P4 dose is sufficient to eliminate short, infertile cycles, enabling effective estrus synchronization and onset of ovarian activity [37]. Females receiving estrus induction protocols supplemented with SeVE achieved 100% induction efficacy [41], likely reflecting an additive or synergistic effect of SeVE on follicular responsiveness and ovulatory outcomes [25,39,42,43].

Regarding ovulation rate—defined as the number of corpora lutea—the SeVE group showed higher values (*p* < 0.05), suggesting that SeVE supplementation during anestrus enhances follicular development and ovulation even with short-term administration [44]. Selenium supplementation has been reported to stimulate ovarian activity by promoting follicular growth and increasing ovulation rate (i.e., corpus luteum formation) [45]. This effect is partly mediated through enhanced cholesterol availability for progesterone (P4) biosynthesis, with the LRP8 receptor facilitating selenium–protein interactions critical for cholesterol homeostasis [46].

Although no direct link between selenium and GnRH has been identified, selenium may influence LH activity by upregulating GnRH receptor expression in the anterior pituitary, potentially increasing LH synthesis [46]. LH promotes ovarian estrogen release, ovulation, and luteal development [47]. Additionally, selenium may enhance ovarian vascularization, contributing to improved follicular and luteal function [48,49]. In cattle, selenium accumulates in granulosa cells of preovulatory follicles and is associated with corpus luteum development [50]. Furthermore, selenium’s antioxidant role protects corpus luteum function by mitigating oxidative damage [51]. Consistent with prior studies, ovarian size did not differ between groups, suggesting that short-term SeVE supplementation impacts ovarian function rather than structural dimensions [50,51,52].

Embryo implantation and pregnancy rates were also significantly higher in the SeVE-supplemented goats. Selenium is known to promote favorable reproductive tract changes that facilitate sperm transit, fertilization, and implantation [53,54]. Selenium enhances uterine architecture and function by reducing epithelial detachment and increasing myometrial mass [55]. Moreover, selenium-induced increases in P4 synthesis correlate with improved endometrial development, conceptus survival, embryo growth, and pregnancy maintenance [25,56,57]. Synchronization between the embryo and the maternal endometrium prior to implantation is critical. Selenium supplementation has been linked to differential gene expression in maternal recognition of pregnancy, particularly those responsive to INFtau and P4 [53]. Using a blend of organic and inorganic selenium (i.e., 35 ppm) has demonstrated improvements in endometrial and conceptus gene expression [58]. Moreover, preventive SeVE supplementation has improved pregnancy rates post-embryo transfer in heifers (64.7% vs. 41.2%); the latter denotes an interesting cross-species relevance in ruminants [59].

Despite these positive effects, some studies report neutral or negative outcomes of selenium supplementation. For example, no differences in reproductive efficiency were observed in some trials [53,54], possibly due to adequate baseline nutrition, which masks supplementation effects. Similarly, no differences in LW or BCS were observed between groups in this study, aligning with prior findings in goats [60,61] and ewes treated with Se and vitamin E during estrus synchronization [25]. In contrast, excessive or long-term selenium supplementation may impair blastocyst quality and implantation success due to tissue selenium accumulation and embryotoxicity [62]. Therefore, assessing baseline feed mineral content before supplementation is crucial, in addition to tailoring interventions to the physiological stage, timing, dose, and administration route to optimize outcomes [63].

Undeniably, the results obtained represent practical and accessible options for goat producers not only from the biological, productive, and economic perspectives, but also in marginal contexts. In fact, the SeVE group showed a 43.8% improvement in the pregnancy rate, which corresponds to an increase in offspring of 0.7 per goat. If the cost per treatment (i.e., SeVE) is equivalent to USD 1.83 per goat and the selling price for a kid is around USD 51.16 (i.e., MXN 1000), revenues increase up to MXN 35.81 per goat. This means that SeVE treatment shows a cost–benefit ratio of 19.57; if producers invest USD 1, they receive USD 19.57. The above considerations are based on the sale of meat alone; such scenarios can rise significantly if additional revenues are derived from an increasing milk volume in dairy goat production systems.

## 5. Conclusions

The obtained results support our working hypothesis that short-term precision supplementation with selenium and vitamin E in adult crossbred dairy goats improves key reproductive variables, such as estrus induction, ovulation rate, embryo implantation, and pregnancy rate. Such enhancements occurred with no significant differences between the experimental groups’ live weight or body condition scores. Similarly, no variations occurred in the diameters of the left and right ovaries or the corpus luteum diameters. Thus, a short-term supplementation may not achieve the requested Se level to potentially affect some “long-term response variables” (i.e., live weight, body condition score, and ovarian size–morphology), but it will undoubtedly affect other “short-term response variables” (i.e., estrus induction (i.e., d27), embryo implantation rate (i.e., d42), and pregnancy rate (d70). Improving reproductive outcomes could help not only to stabilize goat producers’ and their families’ year-round income but could also contribute to higher animal protein supply and greater resilience in marginal goat production systems. Undoubtedly, longitudinal studies to evaluate the economic sustainability and scalability of our research findings, are certainly pending assignments.

## Figures and Tables

**Table 1:** Chemical composition of alfalfa hay, commercial concentrate, and mineral salts that comprised the basal diet in the adult multibreed goats supplemented with selenium + vitamin E (SeVE; *n* = 16) or control (CONT; *n* = 16) under natural photoperiod conditions during the anestrus season in northern Mexico (25° N).

Item	Alfalfa Hay ^a^	Commercial Concentrate ^b^	Mineral Salts ^b^
Nutrient Composition	(%)	(%)	(%)
Dry matter	92.8	- -	- -
Crude protein	15.5	14.0	- -
Neutral detergent fiber	56.9	- -	- -
Acid detergent fiber	41.1	- -	- -
Crude Fat	- -	6.8	- -
Crude Fiber	- -	2.0	- -
Humidity	- -	10.8	- -
Ash	- -	5.5	- -
Nitrogen-Free Extract	- -	62.9	- -
Phosphorus	0.26	- -	9.0
Calcium	1.71	- -	8.5
Magnesium	0.20	- -	1.2
Sodium	0.12	- -	- -
Chlorine	0.35	- -	- -
Potassium	2.16	- -	- -
Iron	- -	- -	740 ppm
Manganese	- -	- -	2030 ppm
Zinc	- -	- -	2600 ppm
Copper	- -	- -	520 ppm
Iodine	- -	- -	96 ppm
Cobalt	- -	- -	12 ppm
Selenium	- -	- -	10 ppm

^a^ Composition values were determined according to the procedures outlined by AOAC, 2023 [34]. ^b^ Nutritional values correspond to the guaranteed analysis declared by the manufacturer.

**Table 2:** Least squares means ± standard error for live weight (LW, kg), body condition score (BCS, units), estrus induction (EI, %), ovulation rate (OR, units), corpus luteum diameter (CLD, mm), left ovary diameter (LOD, mm), right ovary diameter (ROD, mm), embryo number (EMBRYO, units), and pregnancy rate (PREG, %) in adult multibreed goats supplemented with selenium + vitamin E (SeVE; *n* = 16) or control (CONT; *n* = 16) under natural photoperiod conditions during the anestrus season in northern Mexico (25° N).

Variables	SeVE	CONT
**LW, kg**	42.04 ± 1.67 ^a^	41.53 ± 1.43 ^a^
**BCS, units**	1.72 ± 0.06 ^a^	1.66 ± 0.06 ^a^
*Ovarian Size and Activity*		
**EI, %**	80.0 ± 11 ^a^	35.0 ± 12 ^b^
**OR, units**	1.20 ± 0.22 ^a^	0.42 ± 0.22 ^b^
**CLD, mm**	9.1 ± 1.1 ^a^	9.2 ± 1.9 ^a^
**LOD, mm**	16.5 ± 1.2 ^a^	16.8 ± 1.7 ^a^
**ROD, mm**	15.6 ± 1.1 ^a^	17.0 ± 1.5 ^a^
*Embryo Number and Pregnancy Rate*		
**EMBRYO, units**	1.23 ± 0.20 ^a^	0.35 ± 0.19 ^b^
**PREG, %**	75.0 ± 11.4 ^a^	31.2 ± 11.2 ^b^

^a,b^ Values on the same line with different superscripts differ (*p* < 0.05).

## Data Availability

None of the data were deposited in an official repository, yet information can be made available upon request.

## Abbreviations

The following abbreviations are used in this manuscript:

SeVe	Selenium and vitamin E	P4	Progesterone
Se	Selenium	eCG	Equine chorionic gonadotropin hormone
VE	Vitamin E	CONT	Control group
EI	Estrus induction	CL	Comarca Lagunera
OR	Ovulatory rate	DNA	Deoxyribonucleic acid
CLD	Corpus luteum diameter	RNA	Ribonucleic acid
LOD	Left ovary diameter	GSH-Px	Glutathione peroxidase
ROD	Right ovary diameter	CP	Crude protein
EMBRYO	Embryo implantation	Us	Ultrasound
PREG	Pregnancy percentage	SaSo	Saline Solution
LW	Live weight	LH	Luteinizing Hormone
BCS	Body condition score	GnRH	Gonadotropin-releasing hormone
